# A metagenomic-based study of two sites from the Barbadian reef system

**DOI:** 10.1007/s00338-022-02330-y

**Published:** 2023-01-24

**Authors:** S. Simpson, V. Bettauer, A. Ramachandran, S. Kraemer, S. Mahon, M. Medina, Y. Vallès, V. Dumeaux, H. Vallès, D. Walsh, M. T. Hallett

**Affiliations:** 1 Department of Computer Science and Software Engineering, Concordia University, Montreal, Canada; 2 Department of Biology, Concordia University, Montreal, Canada; 3 Coral Reef Restoration Alliance (CORALL), Bridgetown, Barbados; 4 Department of Biology, Pennsylvania State University, University Park, PA USA; 5 Department of Biology and Chemical Sciences, University of the West Indies, Cave Hill, Barbados; 6 Department of Anatomy and Cell Biology, University of Western Ontario, London, Canada; 7 Department of Biochemistry, University of Western Ontario, London, Canada

**Keywords:** Caribbean marine environment, Barbados reef system, Reef microbiome, Whole genome shotgun sequencing

## Abstract

We study the microbiome of sea water collected from two locations of the Barbadian coral reefs. The two sites differ in several environmental and ecological variables including their endogenous benthic community and their proximity to urban development and runoffs from inland watersheds. The composition of the microbial communities was estimated using whole genome DNA shotgun sequencing with adjuvant measurements of chemical and environmental qualities. Although both sites exhibit a similar degree of richness, the less urbanized site (Maycocks reef at Hangman’s Bay) has a strong concentration of phototrophs whereas the more urbanized location (Bellairs reef at Folkstone) is enriched for copiotrophs, macroalgal symbionts and marine-related disease-bearing organisms from taxa scattered across the tree of life. Our results are concordant with previous profiles of warm ocean surface waters, suggesting our approach captures the state of each coral reef site, setting the stage for longitudinal studies of marine microbiome dynamics in Barbados.

**Supplementary Information:**

The online version contains supplementary material available at 10.1007/s00338-022-02330-y.

## Introduction

The capacity of fragile reef ecosystems to maintain key economic, social and environmental services for coastal human societies has been severely challenged by a global decline in coral reef cover of at least 50% (Eddy et al. 2021). The reef system of Barbados (Jackson et al. 2014) and many other small island states in the Caribbean (Burke et al. 2011) have also witnessed comparable coral loss since the 1970s. Documented drivers of coral loss in Barbados include local factors such as sedimentation, eutrophication (Hunte and Wittenberg 1992; Bell and Tomascik 1993) and overfishing (Gill et al. 2019). Regional factors include hurricane damage (Mah and Stearn 1986) and the loss of a keystone grazer (Hunte et al. 1986). Global factors include temperature-induced bleaching due to sea surface warning (Oxenford and Vallès 2016). The relative contribution of these factors to coral loss in Barbados remains unresolved (Wittenberg and Hunte 1992) and likely fluctuates as the reef deteriorates.

The status of the Barbados reef system has been monitored since 1982, providing observational data of widely-used indicators of reef ecological integrity including coral diversity, percent coral and algal cover, and urchin and fish abundance at multiple sites along the west coast of the island (Cermes 2018). However funding constraints limit monitoring to 5 year cycles, a too infrequent interval to inform on reef change in a rapidly evolving global environment, for example, in response to the recent Sargassum invasions (Langin 2018). One motivation of our study here is the need for monitoring techniques that are accessible for small island states such as Barbados (Vallès et al. 2019).

Since microbiomes influence and reflect the environment they inhabit, an understanding of the natural variability and shifts in community gradients of the coral reef waters may provide a more sensitive measure of reef health and allow for the precise identification of environmental disturbances, in turn suggesting prophylactic measures that could be taken to cull negative influences (Glasl et al. 2019; Weber et al. 2020). We profile for the first time the microbiome of reef water at two locations in the Barbadian reef system using whole genome DNA sequencing. These locations were chosen because they lie along a gradient of increasing eutrophication on the west coast of the island and because they have been previously regularly monitored (Bell and Tomascik 1993; Tosic et al. 2009). We ask whether it is possible to identify differences in microbiome composition that reflect differences in eutrophication and other variables that may be used to develop markers which accurately estimate overall reef health, and compare these sites to other oceanic microbial communities (Sunagawa et al. 2015).

## Methods

Water samples were collected from the Bellairs reef at Folkestone Marine Reserve (13°11′30.2″N, 59° 38′ 29.2″ W) and the Maycocks reef at Hangman’s Bay (13° 17′ 32.9″ N, 59° 39′ 47.5″ W) at 13:00–15:00 on January 30 and 31, 2018, respectively. Bellairs is a nearshore fringing reef within the no-take area of Folkstone Marine Reserve in relatively shallow waters (~ 10 m). It lies ~ 100 m from Holetown, a densely populated area with tourism and other urban infrastructure (Helmer et al. 2008). The reef has historically suffered from higher levels of eutrophication (Bell and Tomascik 1993) and episodic run-off during the wet season (Tosic et al. 2009).

**Fig. 1:**
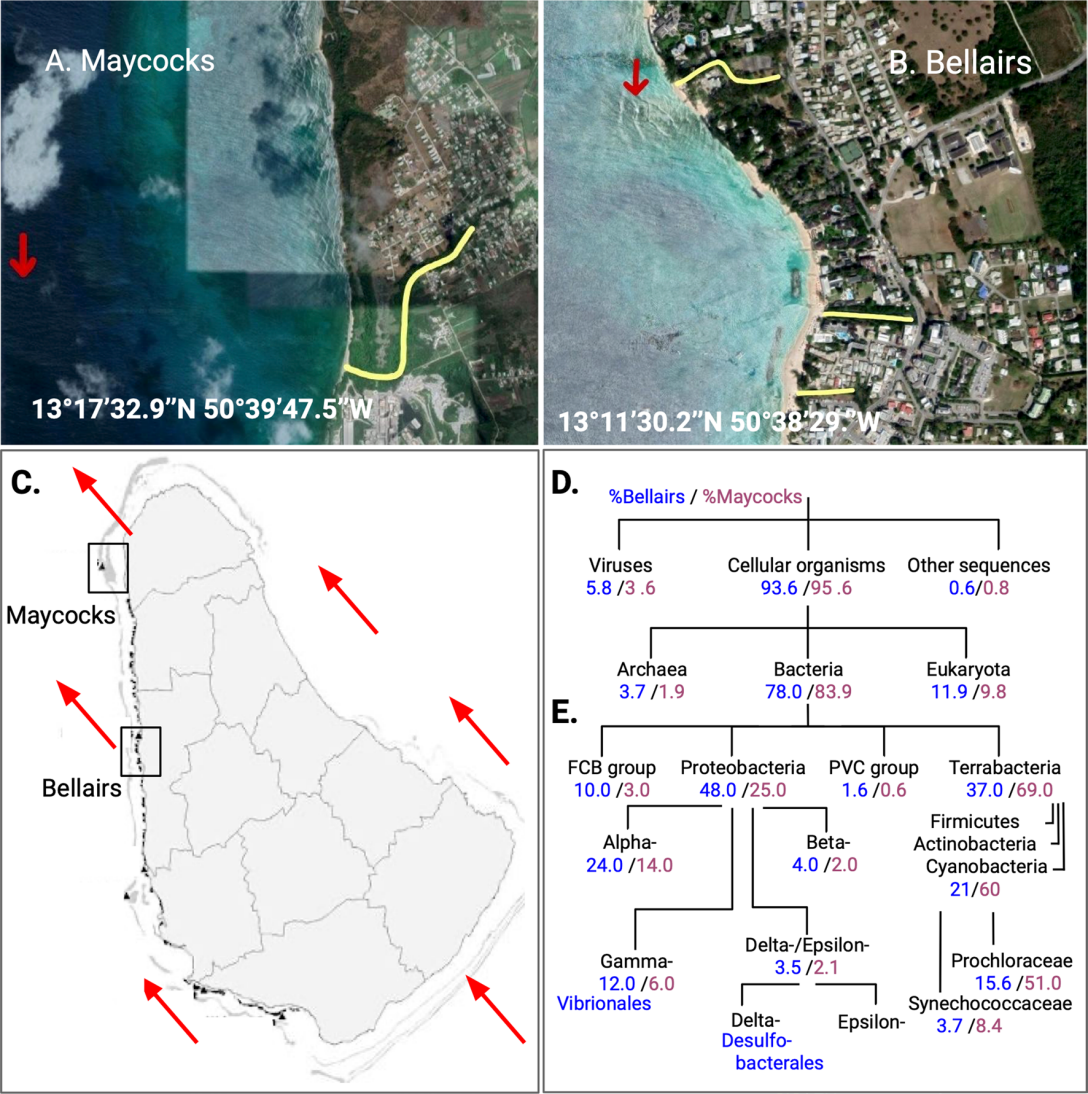
Aerial view of the Maycocks (**A**) and Bellairs (**B**) sites with surrounding areas. Yellow denotes runoffs. **C** Map of Barbados with the sea current direction at time of sampling. **D** Percentage of total read counts at each node relative to its parent. **E** The subtree of Bacteria. FCB refers to Fibrobacteres, Chlorobi, and Bacteroidetes and PVC refers to the superphyum enriched with Planctomycetota, Verrucomicrobiota, and Chlamydiota

Maycocks, which lies north of Bellairs, is a bank reef located in deeper water (~ 20 m) approximately 1.5 km away from a sparsely populated coastline with little coastal development (Fig. 1A, B). This site is subject to relatively low levels of chronic eutrophication (Bell and Tomascik 1993) and is too far offshore to be affected by run-off. Maycocks exhibits a higher abundance and more uniform distribution of corals, significantly more hard corals, less filamentous algae, fewer dead corals and greater average fish biomass (Cermes 2018) (Supplemental Table 1A).

At time of sampling the ocean current was flowing in a northwestern direction (Fig. 1C). Sea water was collected at ~ 1 m above the reefs. For each sample (2 per location), 7 L of seawater was sequentially filtered through a 50 μm pore mesh, followed by filtering to discard organisms larger than 3 μm and smaller than 0.22 μm (polycarbonate Sterivex filter, Durapore; Millipore Inc.) consistent with the Tara Oceans project (Sunagawa et al. 2015). Filters were preserved in RNAlater at − 80 °C until processing. DNA was extracted from the Sterivex filter after thawing on ice and RNAlater was removed. The filters were rinsed twice with a sucrose-based lysis buffer and given 1.8 mL of lysis buffer. Filters were then treated with 100 μL of 125 mg mL^−1^ lysozyme and 20 μL of 10 μg mL^−1^ RNAse A and left to rotate at 37 °C for 1 h. Then, after incubation, 100 μL of 10 mg mL^−1^ proteinase K and 100 μL of 20% SDS were added. Filters were rotated for 2 h at 55 °C and the lysate removed. Protein was then precipitated and removed with 0.583 volumes of MPC protein Precipitation Reagent (Epicentre Inc.) and centrifugation at 10,000 × g at 4 °C for 10 min. The supernatant was transferred to a clean tube. The DNA was precipitated with cold isopropanol and resuspended in low TE buffer.

For whole genome sequencing, DNA was prepared (Nextera DNA Library Kit FC-131-1086, Illumina Inc.) and sequenced on an Illumina NovaSeq PE 150 platform (2 × 150 bp reads). Kraken2 version 2.1.0 (Wood and Salzberg 2014) and Bracken 2.6.0 (Lu et al. 2017) were used for alignment and classification with NCBI downloads (restricted to all single cell organisms), the MAR reference database (Klemetsen et al. 2018) and the NCBI Taxonomy database (3/31/2020). Reads mapped to non-microbial taxa were removed from this study. The final microbial dataset after filtering and quality control consists of 4.5 M and 9.2 M reads at Bellairs and Maycocks, respectively (Supplemental Table 1C). Oxygen, seawater temperature and salinity were measured with an EXO2 multiparameter sonde (YSI Inc.; 3 × each per site). Nitrates were measured using the cadmium reduction method with a HACH NitraVer 5 nitrate reagent (3 reps); nitrites were measured using the diazotization method (3 reps; HACH NitriVer 3 nitrite reagent); phosphates were measured using the ascorbic acid method (3 × reps; HACH PhosVer phosphate reagent). All readings were performed with a HACH DR3900 spectrophotometer. Turbidity was measured using a HACH 2100P turbidity meter (3×). Counts of organisms were generated via epifluorescence microscopy as described in (Dinsdale et al. 2008).

The Kruskal–Wallis (KW) test was used to measure significance between the frequencies of child taxa (e.g., genera) within a specific ancestral taxa from the first site versus the frequencies of these same child taxa at the second site. When the KW test was significant, Dunn’s test was used to determine which specific child taxa were significantly different. In some instances we asked if the distribution of observed genera were enriched in a specific ancestral taxa (e.g., clades or phyta); towards this end we used the hypergeometric test where bins correspond to the ancestral taxa and bin size was obtained from our NCBI database above. Clustering was performed by first identifying the most abundant taxa for each sample (Supplemental Information 4), and then transforming their abundances to ranks before use of the Kendall τ distance metric with Ward’s algorithm to construct two dimensional hierarchical clusters.

## Results and discussion

We found striking and consistent differences between Bellairs and Maycocks in many dimensions. Maycocks had higher concentrations of dissolved oxygen whereas Bellairs had higher concentrations of nitrate (Supplemental Table 1B). In total, 97% of the genera were identified at both sites through analysis of the sequencing data (Supplemental Table 1C and Supplemental Information 1 for richness analyses). However, the relative frequency of taxa is very different between the two sites. For example, Maycocks exhibits a relative enrichment of Bacteria and Chlorophyta whereas Bellairs contains more eukaryotic (primarily fungal) and archaeal taxa (Fig. 1D; all *p* << 0.01, Pearson’s $\chi$^2^). Then within the bacterial superkingdom, there are more pronounced differences in the distribution across phyla (Fig. 1E). For example, Maycocks has a strong preference for the Terrabacteria group (37% B vs 69% M; KW test, *p* << 0.01), whereas Bellairs has a preference for Proteobacteria, specifically the Alpha class. We explore these differences below.

**Fig. 2:**
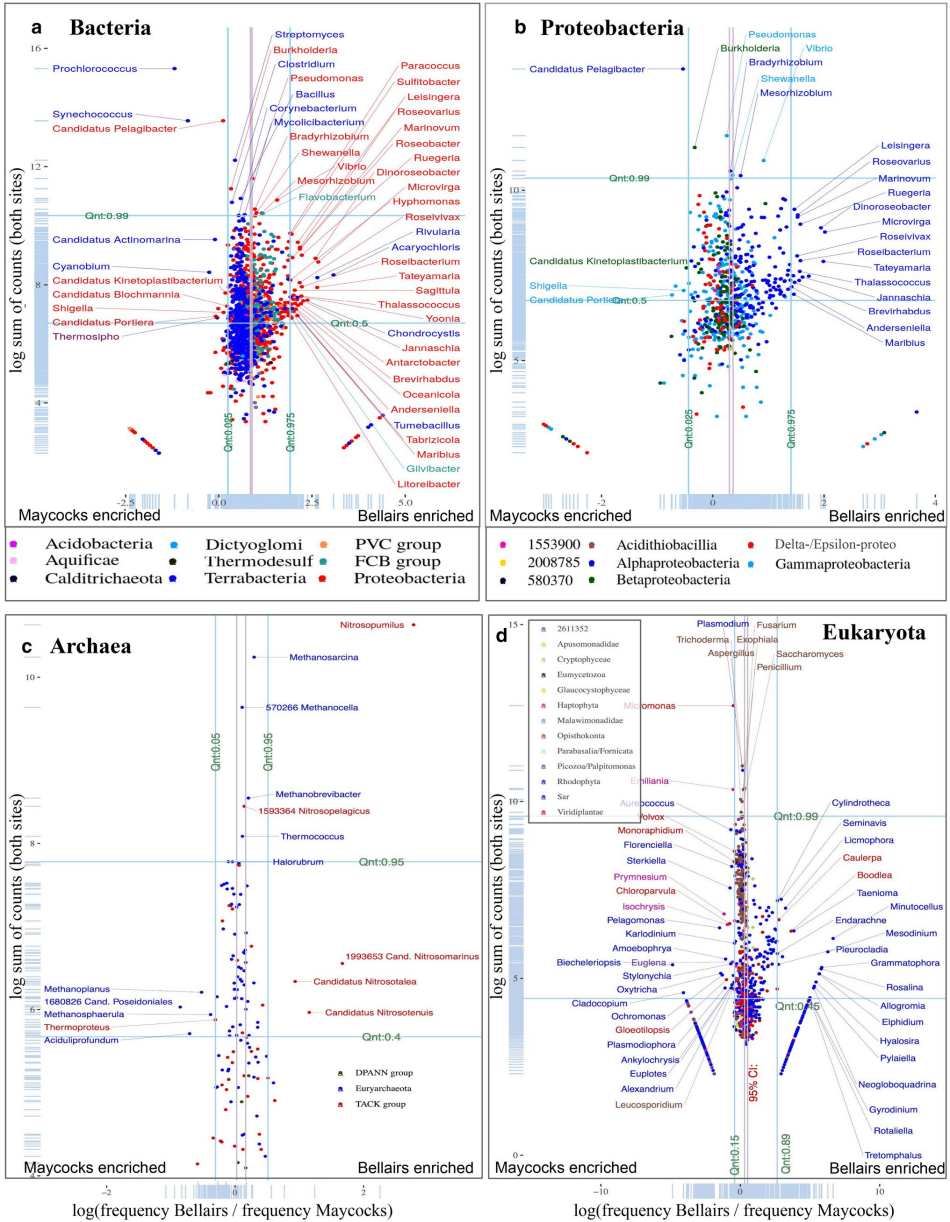
**A** Here the x-axis is the log ratio of the fraction of all bacterial reads at Bellairs versus the fraction of all bacterial reads at Maycocks. *Y*-axis is the log of the sum of all reads across all genera within Bacteria, a measure of overall abundance. Light blue tickets on margins represent a rug plot to indicate distribution of genera across axes. This is done at the level of genera. **B**–**D** are analogous to panel A except for Proteobacteria, Archaea, and Eukaryota respectively. Vertical red lines indicate a 95% confidence interval the location of the mean. Blue lines indicate a 95% confidence interval on the distribution of the log-ratio for the genera. Light blue margin ticks represent a rug plot to indicate density distribution of genera along axes

*Maycocks is highly enriched for phototrophic microbes* including the autotrophic genera Prochlorococcus and Synechococcus which account for over 98% of all Cyanobacteria identified in our study, a fact consistent with the oligotrophic nature of the Barbadian marine environment (Biller et al. 2015). This is consistent with our microscopy imaging which suggests that Maycocks has a tendency towards smaller organisms (Supplemental Fig. 1). Prochlorococcus is typically more prevalent in nutrient poor water while Synechococcus is more prevalent in eutrophic water and coastal plumes of rivers (Wawrik et al. 2003). This is consistent with our data where we observe a 3.7:1 Prochlorococcus to Synechococcus ratio at Bellairs, but a 5.3:1 ratio at Maycocks. The observed frequencies of both genera at Maycocks are more extreme than all other locations world-wide, including nearby Curacao (Weber et al. 2020). Candidatus Pelagibacter, which belongs to the ubiquitous marine SAR11 clade, is also significantly shifted towards Maycocks and is one of the most abundant genera identified in our study (KW test, *p* << 0.01, Fig. 2A). These small free-living heterotrophic species thrive in low-nutrient environments and play a significant role in carbon cycling (Dinasquet et al. 2019).

The enrichment of phototrophs at Maycocks also holds in the Archaea superkingdom. This includes euryarchaeota such as Candidatus Poseidoniales, which contains some of the most abundant planktonic archaeons in ocean surface waters. It is a motile photoheterotroph capable of degrading proteins and lipids (Rinke et al. 2019) (Fig. 2C). Although the two sites have a similar percentage of Eukaryota, it is the Viridiplantae which exhibit the largest enrichment at Maycocks (21% B vs. 32% M). Some 99% of these reads map to the autotrophic green algae Chlorophyta (Fig. 2D). This includes Micromonas pusilla and M. commoda which are photosynthetic picoeukaryotes known to thrive globally in tropical marine environments and play a key role in the primary production within the euphotic zone (Šlapeta et al. 2006).

*Bellairs is enriched for copiotrophs* from taxa scattered across the tree of life, an observation consistent with its proximity to urban development and inland run-offs. There is a significant enrichment of Proteobacteria at Bellairs (Fig. 2A), with emphasis on the Alpha class which are major contributors of bacterioplankton to ocean waters (Dunn’s test, *p* << 0.01; Fig. 2B). The majority of genera within Rhodobacteraceae show a general trend towards the Bellairs site (KW test, *p* << 0.01), highlighting Dinoroseobacter, Tateyamaria and Jannaschia. Dinorosebacter are aerobic anoxygenic phototrophic bacteria, highly abundant in marine turf algae. Tateyamaria is a genus of coastal aerobic bacteria which can thrive under acidification conditions (Huggett et al. 2018). Jannaschia are aerobic anoxygenic phototrophic bacteria and some species play a role in nitrate reduction (Moran et al. 2007). Bellairs is also slightly enriched for several species of the highly abundant Vibrio genus, of which many species play a significant causative role in coral diseases (Munn 2015) (Supplemental Information 2).

**Fig. 3:**
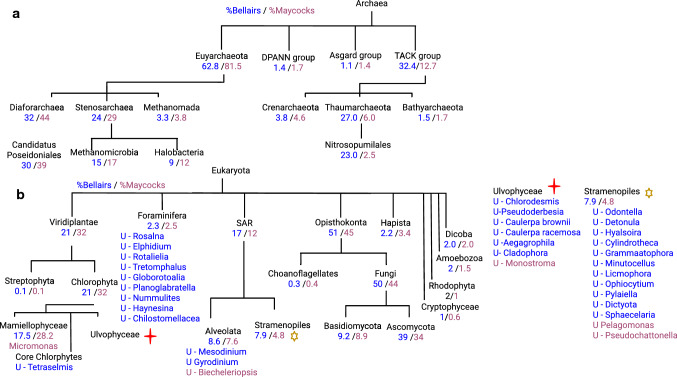
Analogous Fig. 1E except for (**A**) Archae and (**B**) Eukaryota respectively. The Foraminifera, Stramenopiles (yellow star) and Ulvophyceae (red cross) are statistically enriched for the vast majority of genera identified only at Bellairs (blue) or Maycocks (red) sites; genera for the latter two are listed on the right-hand side

With respect to Archaea, Bellairs is strong enriched for thaumarchaeota involved in nitrification. (23% B versus 3% M, Dunn’s test, *p* << 0.01; Fig. 2C, Fig. 3A). The chemolithoautotrophic Nitrosopumilus maritimus species is a dominant contributor to nitrification in marine environments (Sánchez-Quinto and Falcón 2019) and Candidatus Nitrosopelagicus is a planktonic pelagic ammonia-oxidizing thaumarchaeon involved in nitrogen and carbon fixation (Santoro et al. 2015).

With respect to Eukaryota, Bellairs is enriched for benthic epiphytes growing on coral and algae. Many species of Stramenopiles within the SAR clade are observed uniquely at Bellairs (Fig. 3B denoted by a yellow star, 11 of all 34 uniquely identified organisms identified at Bellairs (hypergeometric binned by eukaryotic phyta, *p* << 0.01). This is not likely due to the depth of sequencing alone given that Maycocks in fact received 1.57 fold more reads than Bellairs and analysis in Supplemental Information 1). Several marine diatoms were identified including Licmophora, Cylindrotheca, Seminavis, and others which contribute species that grow on algae and corals or feed on diatoms in eutrophic environments and are causal in brown and red tides. Supplemental Information 3 provides a more detailed investigation of the SAR clade. Analogous to the Stramenopiles, the Foraminifera contribute a surprising number of uniquely identified genera (9 of 34; *p* << 0.01, hypergeometric binned by clades; Fig. 3B).

**Fig. 4:**
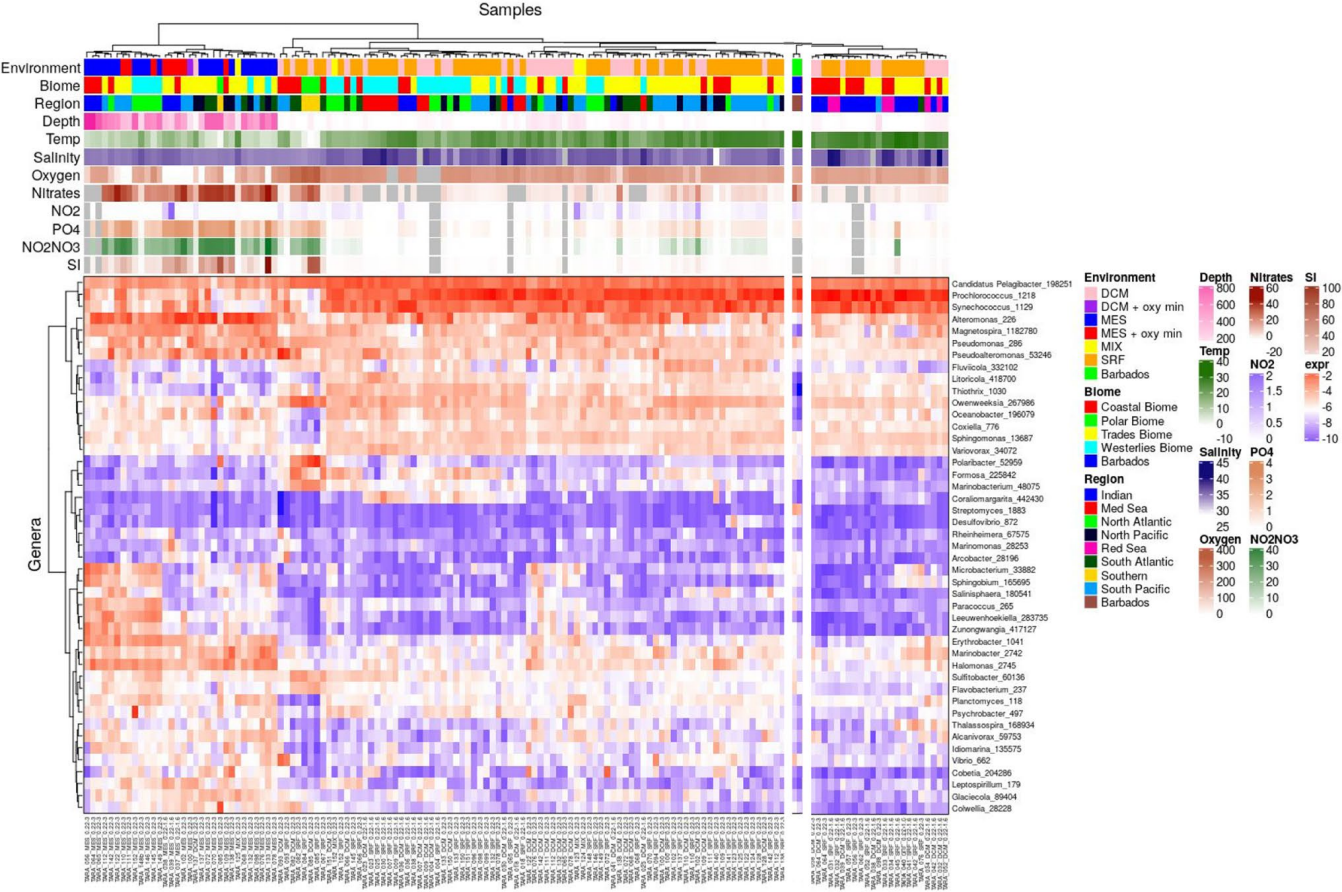
A heatmap of relative abundance of individual species, and associated measures of ecological diversity and physio-chemo-hydrographic attributes integrating the Barbadian coral reef profiles with the Tara Oceans’ data. DCM abbreviates deep chlorophyll maximum; SRF abbreviates surface water; MES abbreviates mesopelagic water

We asked how our sites compare to other ocean waters previously profiled by the Tara Oceans (*N* = 68 epipelagic and mesopelagic locations) (Sunagawa et al. 2015). After normalizing our data with Tara Oceans’ and applying unsupervised clustering of genera frequencies, we see that both sites co-cluster with samples harvested from the surface and deep chlorophyll maximum layers (SRF, DCM respectively) of the trade and coastal biomes (Fig. 3C). This is consistent with the finding from Sunagawa et al. that depth is the most important factor that determines species abundance. Both Bellairs and Maycocks are closest to Red Sea and Indian Ocean samples. Although concentrations of nitrates NO_2_ and PO_4_ at Maycocks are similar to levels of its neighbors in the clustering, nitrate levels of Bellairs are higher and more similar to mesopelagic layers. The right subtree of Fig. 4 is enriched for autotrophs consistent with the hot, oligotrophic waters of Barbados.

Although it is infeasible to draw definitive conclusions from only two sites, the microbiome-level profiles are consistent with a shift from coral to macroalgae between Maycocks and Bellairs. This observation is further supported by our chemical/environmental and microscopy data, in addition to documentation of the benthic communities and with known differences in stressors between the two sites. However, our microbiome approach here provides potentially more mechanistic insight into the global effects of some stressors (e.g., run-off) on the state of reef health than standard monitoring efforts (e.g., benthic communities), as it identifies ~ 100 distinct differentially abundant genera which may interact with different dimensions of the reef ecology. As such, the findings here may influence, for example, the design and feasibility of coral rehabilitation projects and, more generally, our capacity to identify microbiome changes consistent with known stressors and states of eutrophication. This sets the stage for systematic studies using microbiome-based markers of the rate of change to reef health along many variables (e.g., geographic location, degree of urbanization, seasonal change). Our approach here should have relevance to other small islands in the vicinity of Barbados with similar reef systems and stressors.

## Supplementary Information

Below is the link to the electronic supplementary material.

Supplementary file1 (DOCX 4509 KB)

## Data Availability

Raw sequencing data is available at NCBI SRA accession PRJNA685579. Normalized data is available at JGI GOLD with study ID Gs0136136. Code is available via GitHub https://github.com/hallettmiket/barbadian-reef which links to data, code and analyses at Zenodo.
