# Supplementary material for: A metagenomic-based study of two sites from the Barbadian reef system
Source: Coral Reefs. 2023 Jan 24;42(2):359–66. doi: 10.1007/s00338-022-02330-y (PMC10060342; doi:10.1007/s00338-022-02330-y)
Supplement: Supplementary file 1 — Supplementary file1 (DOCX 4509 KB) [file 338_2022_2330_MOESM1_ESM.docx]

**Supplemental Information:** **A metagenomic-based study of two sites from the Barbadian reef system**

Simpson S^1^, Bettauer V^1^, Ramachandran A^2^, Kraemer S^2^, Mahon S^3^, Medina M^4^, Vallès Y^5^, Dumeaux V^6^, Vallès H^5^, Walsh D^2^, Hallett MT^7^

^1^ Department of Computer Science and Software Engineering, Concordia University, Montreal, Canada

^2^ Department of Biology, Concordia University, Montreal, Canada

^3^ Coral Reef Restoration Alliance (CORALL), Barbados, West Indies

^4^ Department of Biology, Pennsylvania State University, University Park, PA, USA

^5^ Department of Biology and Chemical Sciences, University of the West Indies, Cave Hill, Barbados, West Indies

^6^ Department of Anatomy and Cell Biology, University of Western Ontario, London, Canada

^7^ Department of Biochemistry, University of Western Ontario, London, Canada

| **A. Benthic community** | | |  | **B. Chemical and Environmental** | | |  | **C. Sequencing parameters** | | |
| --- | --- | --- | --- | --- | --- | --- | --- | --- | --- | --- |
|  | Bell. | May. |  |  | Bell. | May. |  |  | Bell. | May. |
| Gorgonians | 0.14 | 0.83 |  | Dissolved Oxygen (%) * | 89.73  sd 0.95 | 95.30  sd 0.2 |  | Paired-  end reads | 18.6M | 29.1M |
| Hard Coral | 15.85 | 26.94 |  | Dissolved Oxy. (mg/L) * | 5.68  sd 0.08 | 6.04  sd 0.03 |  | Reads  classified | 7.4M | 13.1M |
| Sponges | 11.50 | 20.99 |  | Salinity (ppt) | 35.03  sd 0.48 | 34.78  sd 0.60 |  | Metazoan reads | 1.7M | 2.3M |
| Filamentous algae | 54.84 | 26.86 |  | Temp (°C) | 29.03  sd 0.01 | 29.13  sd 0.05 |  | Embryo-  phyta | 1.1M | 1.6M |
| Coralline algae | 13.32 | 22.48 |  | Nitrate (NO_3_) (mg/L) ** | 1.78  sd 0.35 | 1.06  sd 0.21 |  | Microbial  reads | 4.5M | 9.2M |
| Sand  Rubble | 4.0 | 0.99 |  | Nitrite (NO_2_)  (mg/L) | 0.018  sd 0.0 | 0.013  sd 0.00 |  | Taxa | 14.6K | 15.2K |
| Filamentous  cyanobac. | 0.21 | 0.83 |  | Phosphate (PO_4_) (mg/L) | 0.070  sd 0.04 | 0.078  sd 0.08 |  | Genera | 2415 | 2479 |
| Zoanthid | 0.14 | 0.08 |  | Turbidity (NTU) | 6  sd 2.65 | 7  sd 1.00 |  | Species | 9089 | 9461 |

**Supplemental Table 1. Comparison of the Bellairs and Maycocks sites across different parameters. A**. The benthic composition of the Bellairs and Maycocks Reef based on the percentage cover of major benthic categories on the substratum (Oxenford and Valles, 2016). **B.** Measurements of several environmental and chemical variables. The * and ** denote significance at p < 0.01 with a t-test and Wilcoxon test respectively. **C.** Results related to the whole genome metagenomic sequencing.


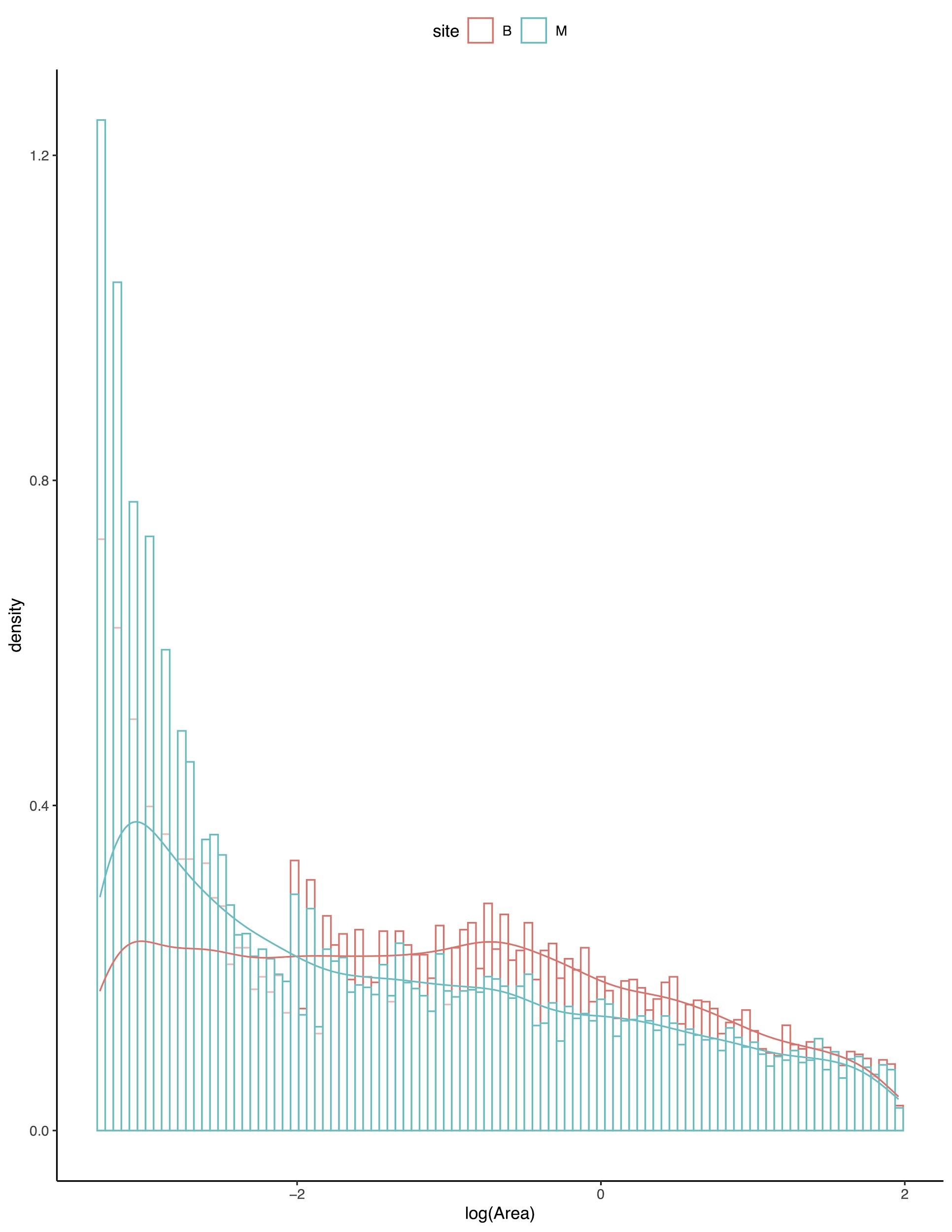


**Supplemental Figure 1**. Distribution of the (log) area of each detected organism across ~270K field of views (FOVs) from 60 images of reef water from Bellairs and Maycocks. We observe an enrichment of small organisms at Maycocks (blue).

**Supplemental Information 2: Richness, diversity, handling zeros and correcting for genome size**

Our metagenomic data is compositional in nature (Aitchison 1986; Gloor et al. 2017; Quinn et al. 2019) and we have followed so-called CoDa (Compositional Data) best-practice guidelines (Gloor et al. 2017; Quinn et al. 2019).

Biodiversity can be measured in many different ways including taxonomic diversity (the presence of different species), phylogenetic diversity (the presence of different evolutionary lineages), or functional diversity (the variety of growth forms and resource use strategies)[^2^](https://www.zotero.org/google-docs/?M9JxFw). *Abundance* refers to the fraction of each species at a site, and since we are in CoDa setting, this is inherently a relative abundance. Diversity is a measure of the distribution of these abundances. Throughout this manuscript we use the Shannon index as our measure of diversity[^3^](https://www.zotero.org/google-docs/?Vsm5Wa) with a permutation based approach to estimate p-values

$$H' = {-\Sigma}_{t \in T}p_{t} ᐧ ln p_{t}$$

where $T$is the vector of relative abundance for all taxa and $p_{t}$is the probability of taxa $t$. Briefly, there is an increase in the Shannon index (entropy) as the distribution of abundances approaches a “flat” uniform distribution, and decreased entropy as the frequency of one (or a few species) approaches one.

*Richness* is defined as the number of species within a specified clade at a site. We asked if there was a difference in richness between Bellairs and Maycocks. However, we must first adjust for the fact that Maycocks and Bellairs had different levels of sequencing coverage. More specifically, budgetary and technical limitations imply that the number of draws made by the sequencer from the urn is finite although large. The sequencing coverage (total number of reads) may not be sufficiently large to identify with high probability rare species in the sample. For instance, a species whose DNA contributes only 1 read to an urn with 10M reads is unlikely to be identified, if the sequencing coverage is only 1M. In our data, we obtained ~4.5M and ~9.2M mappable paired-end reads from Bellairs and Maycocks respectively. Therefore, we expect to identify more taxa in Maycocks than in Bellairs due to this reason alone. In other words, species richness increases with sample size, and differences in richness may be due to differences in sample size. To address this, we first downsampled from the Illumina paired-end reads from 1-50%, and counted the number of identified species and genera (**Supplemental Figure 2**). After only ~3M reads at Maycocks and ~1.5M reads at Bellairs (<⅓ of total in both cases), all taxa have been identified. Maycocks however converges 272 more species (64 more genera) than Bellairs.

Rarefaction provides a second approach to exploring this issue. A statistical correction is computed that estimates the number of taxa we would have observed at the sites if we had sequenced Maycocks to the same coverage as Bellairs. To rarefy samples from N to n total reads, we used the rarefy function in the vegan package[^4^](https://www.zotero.org/google-docs/?aGsaR7). In particular, we extrapolated the number of species at each site individually and also with pooled data using the prestondistr function to estimate the log-normal fit, and the veilespec function to estimate the integral fitted with prestondistr. Consistent with your downsampling approach, this statistic suggested that no change in the number of identified species. This suggests our sequencing coverage is sufficient for all but the most extremely rare organisms.

In ecological communities including marine, most species are rare[^5^](https://www.zotero.org/google-docs/?sJrW4f). Preston argued that this implies that richness would follow a truncated log normal distribution (Preston 1948). This is true for the relative frequencies obtained for our data as depicted in **Supplemental Figure 3**. This allows us to estimate the theoretical richness at both sites, the so-called Preston veil[^4,6^](https://www.zotero.org/google-docs/?ZQtDOw). Specifically, by integrating the fitted log-normal, the Preston veil speculates how long the right tail is if we had infinite sequencing data. However, consistent with the analysis above, the Preston veil did not predict any new species would have been identified (it predicted 0.28 more species above the 9089 species observed). The results for Maycocks were equally insignificant.

If there is a large difference in the sequencing coverage between sites (as is our case), the presence zeros in the shallower site can have non-intuitive effects on analyses especially with respect to distance measures and clustering. Moreover, CoDa analysis (such as ours here) often relies on log-ratios of the form $log ( \frac{p_{B, t}}{p_{M, t}})$ where $p_{B,t}$and $p_{M,t}$are the estimates of the frequency of taxa $t$ at Bellairs and Maycocks respectively. This is referred to as the zero-handling problem[^7^](https://www.zotero.org/google-docs/?f0aBuK). We applied a Bayesian-multiplicative replacement strategy that adjusts the count matrix (for all taxa at both sites) in a manner that preserves the ratios between the non-zero components[^8^](https://www.zotero.org/google-docs/?SBM712). However, when external datasets (eg Tara Oceans) were included in this analysis with lower sequencing coverage, they had > 50% zeros. We were not able to reach convergence unless we removed taxa. After removing taxa that had zero counts in more than 50% of the samples, we reached convergence, but the adjustment had little effect on the data.

*Correcting for genome size.* We asked if there was a correlation between genome size and number of reads aligned to each species across Archaea, Bacteria, Eukaryota and Viruses. The log-log scatterplots of **Supplemental** **Figure 6** depict these relationships across all species. Visual inspection suggests a correlation between log genome size and log read count as expected. To adjust for this effect, we fit a simple linear model of the form

$$log (f_{s,t}) ∽ log (g_{s}) + \varepsilon$$

where f_s,t_ is the fraction of all reads mapped to taxon t at site s, and g_s_  is the genome size (Mbp) for species s, and ε is a normally distributed random variable. An implicit assumption in this simple model is that the vast majority of taxa have approximately the same fraction of read counts f_s,t_. The parameters of the fit were then used to correct the observed read counts. Given the compositional nature of our data, any investigations in this manuscript that seek to compare two taxa *within* the same site must first adjust read counts using the linear model.

The corrected relative abundance estimates of all species are depicted in **Supplemental Figure 7**. The adjustments highlight a high abundance of the bacteria Candidatus Pelagibacter and Prochlorococcus. Several Archaea including the Marine Group II/III and Cand. Poseidoniales appear on magnitude below, followed by viruses that inflect Prochlorococcus and lastly one the Eukaryota Micromonas commoda appear.


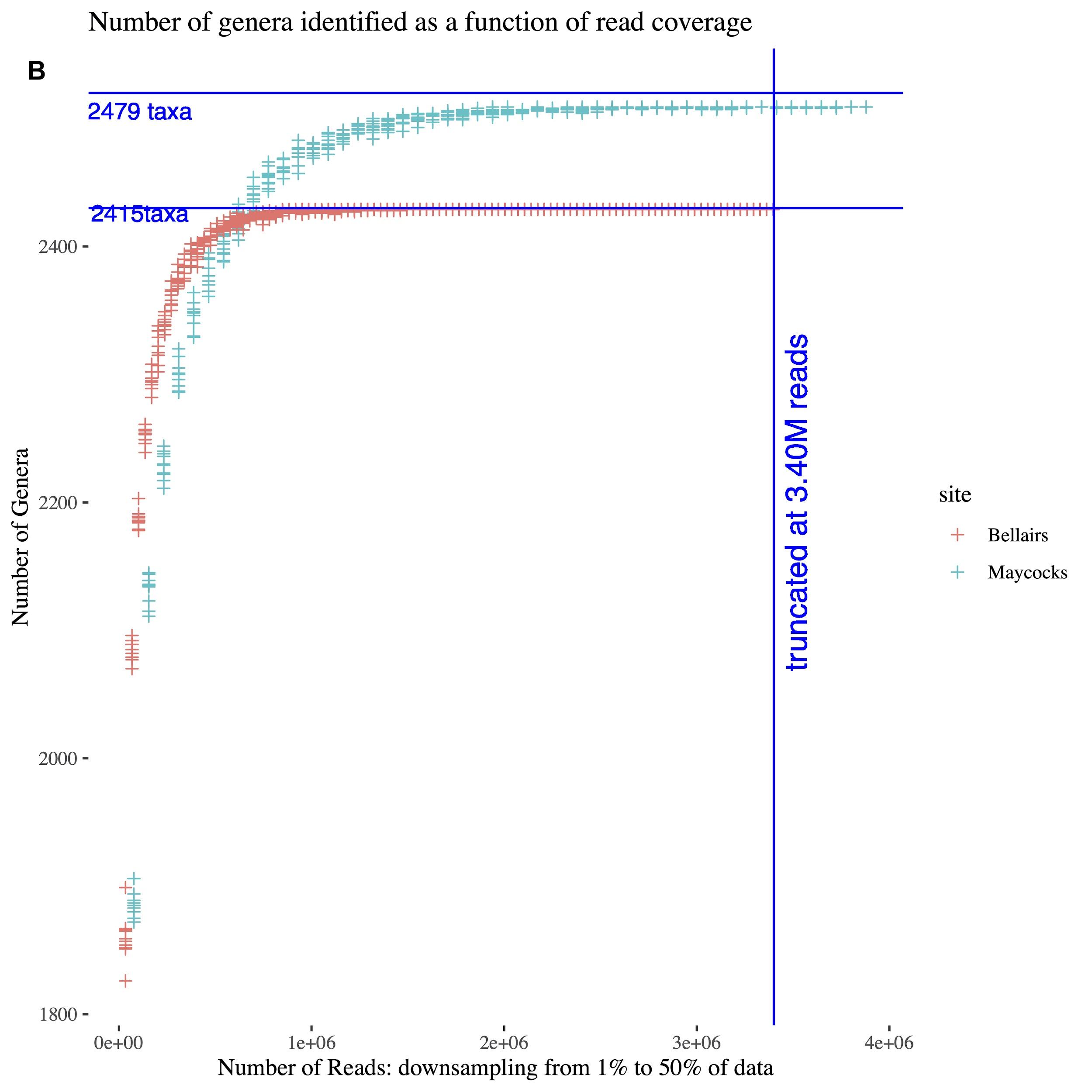

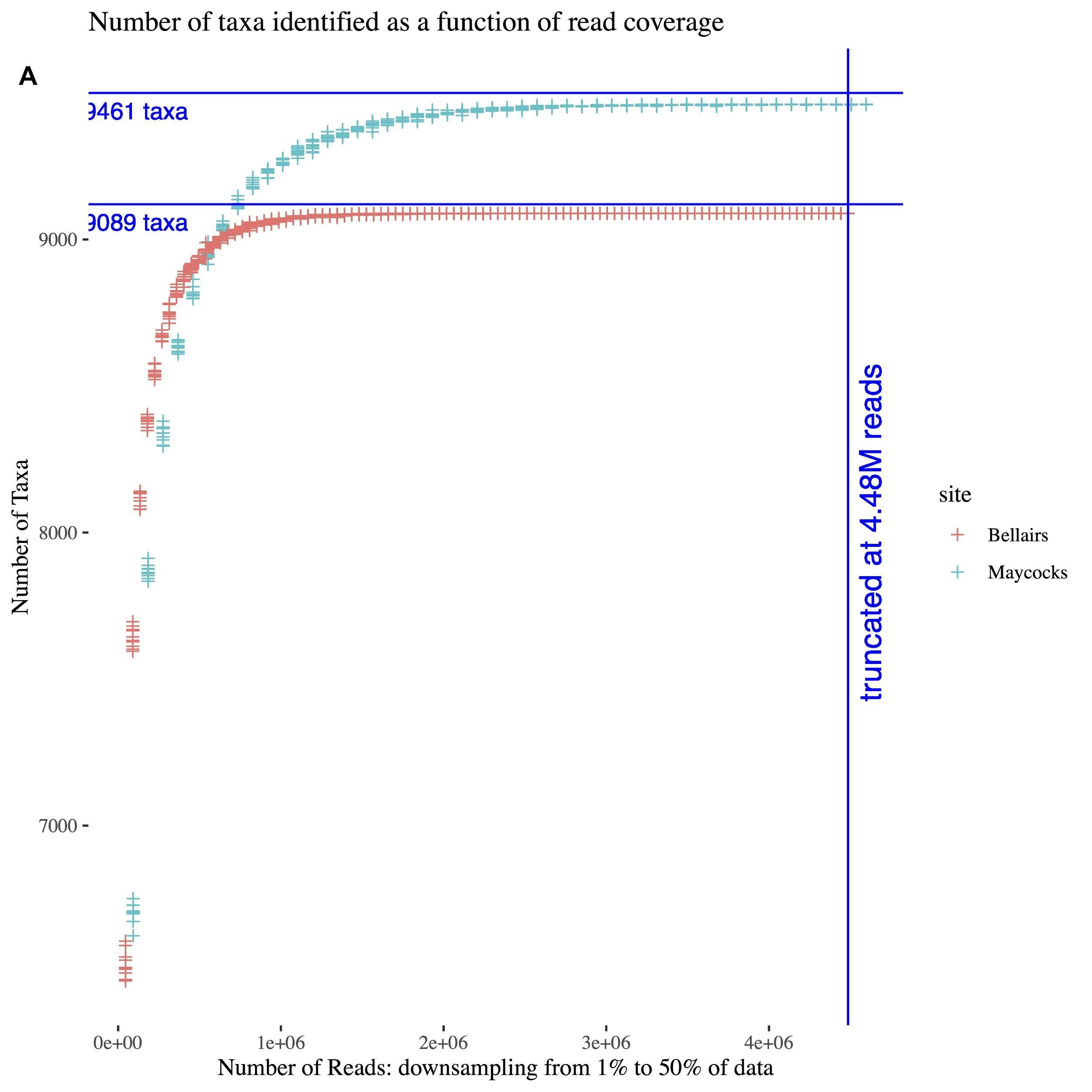


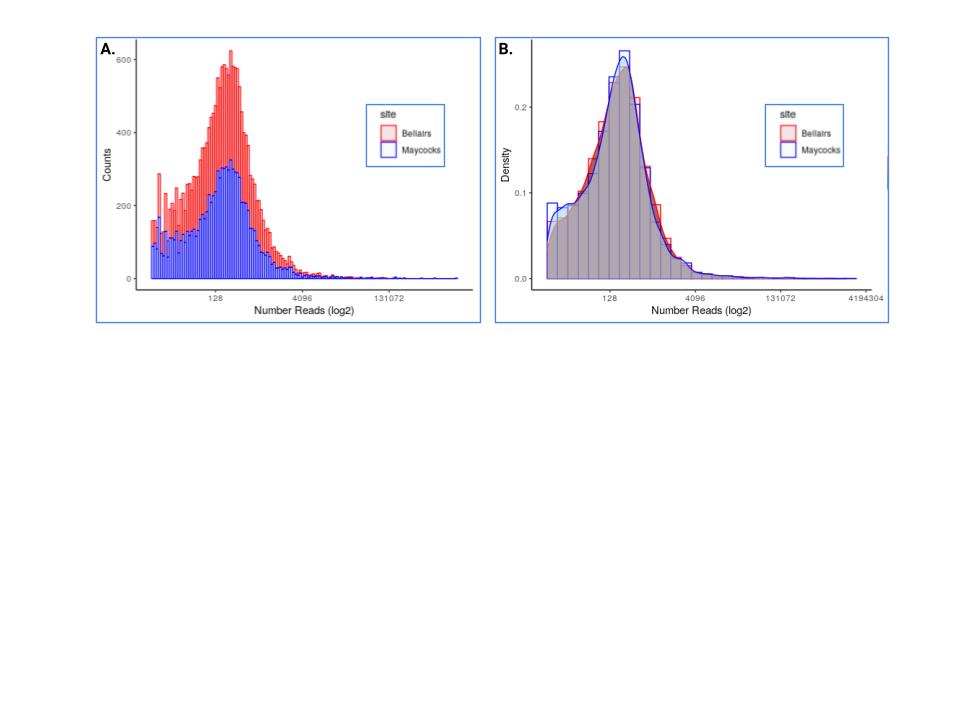
**Supplemental Figure 2 A.** Downsampling of the paired-end reads highlighting the number of species identified. **B.** Downsampling of the paired end-reads highlighting the number of genera identified.

**Supplemental Figure 3 A** The estimated number of species at both sites versus the log number of reads. **B** is the corresponding histogram of frequencies.


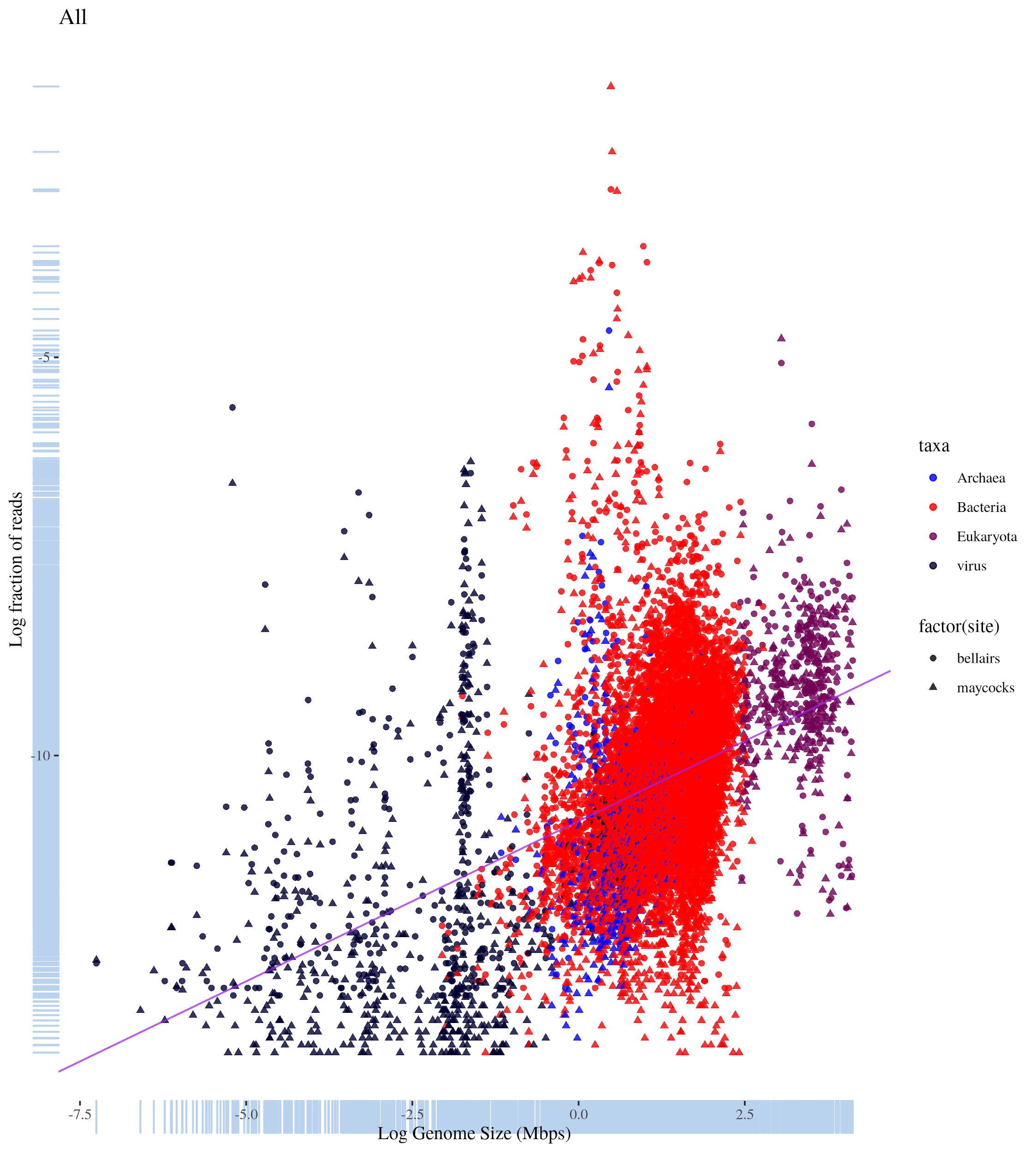


**Supplemental Figure 4.** The relationship between genome size and number of reads mapped to the genome across all domains before correction.


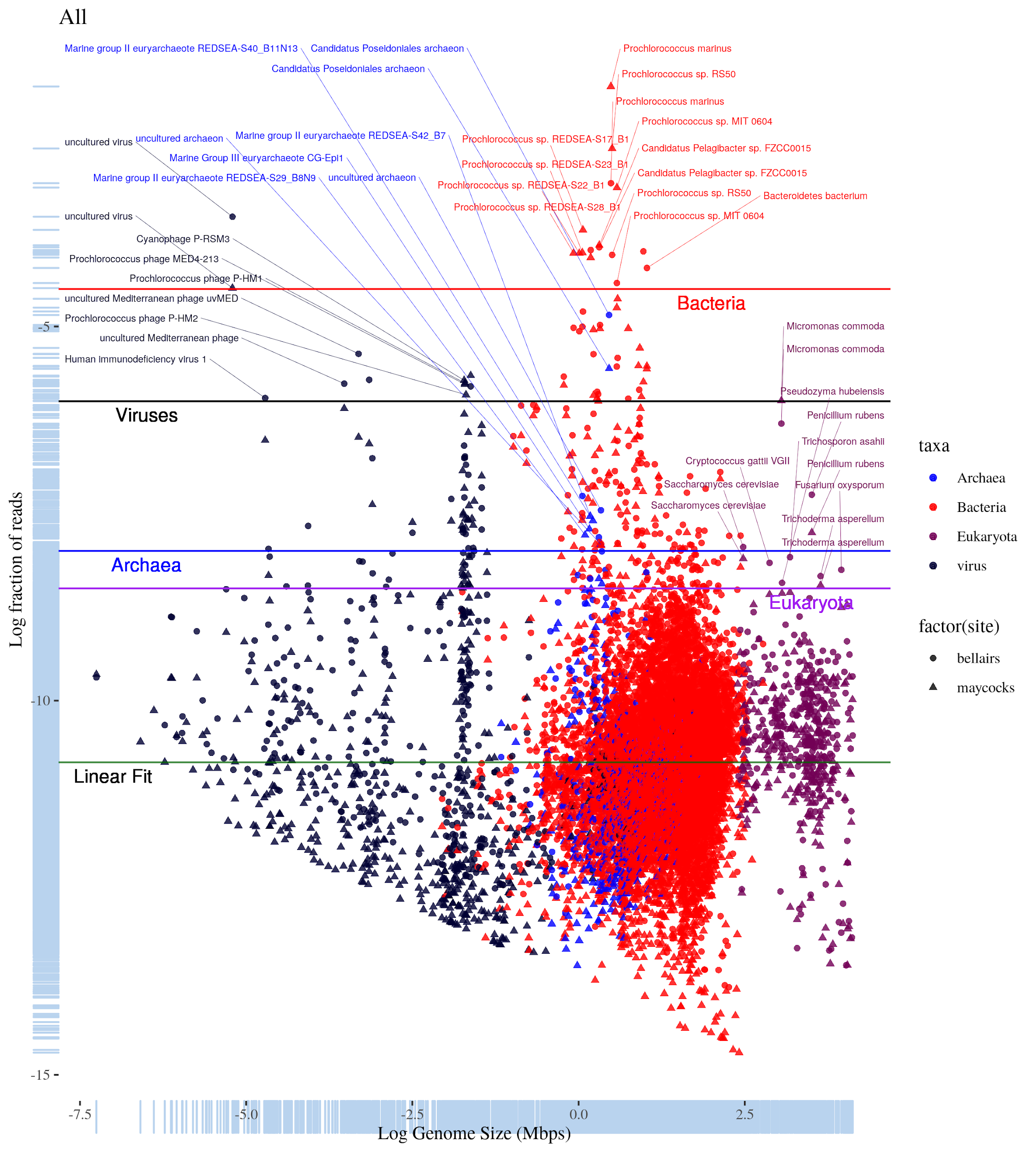


**Supplemental Figure 5.** The relationship between genome size and number of reads mapped to the genome across all species across and all domains after adjusting by the slope obtained from a linear model across all species.

**Supplemental Information 2: the genus Vibrio at Bellairs**

Within Gammaproteobacteria, the genus Vibrio was one of the most highly abundant genera in our dataset and exhibits a strong skew towards Bellairs (KW, p << 0.01) involving many species including V. corallilyticus, V. tubiashii, V. harveyi, V. astriarenae, V. nigripulchritudo, and V. ponticus (**Supplemental Figure 6**). Vibrio are gram-negative bacteria that are motile and found in marine environment; they are facultative anaerobes, capable of aerobic respiration or fermentation depending on whether oxygen is present or not respectively. Some Vibrio species play a significant causative role in coral diseases and disrupt corals symbiotic relationship with zooxanthellae. There are indications that high nutrient levels promote pathogenic bacteria including Vibrio spp. to dominate in healthy coral reef systems (Munn 2015). In our data, there was evidence for several Vibrio species at Bellairs including V. corallilyticus (implicated in white band syndrome) and V. harveyi (linked to yellow spot syndrome but also present in healthy corals albeit less frequently (Cróquer et al. 2013)), V. tubiashii (implicated in shellfish vibriosis and may also be a virulence factor in diseases of scleractinian corals, where it plays a role in photoinactivation of the coral(Sussman et al. 2009)), V. astriarenae (a generalist in many reef systems (Amin et al. 2016)), V. nigripulchritudo (a shrimp pathogen with major impact on farms in Japan and New Caledonia (Goarant et al. 2006)), and V. ponticus (a fish pathogen (Xie et al. 2007)). Photobacterium, also a genus of Vibrionales, is common in marine environments and can survive in both aerobic and anaerobic environments. P. damselae, which was more abundant at Bellairs, is a well-studied pathogen of marine organisms including fish and has made significant negative financial impact on fisheries world-wide (Rivas et al. 2013). Two species of the genus Acinetobacter within Pseudomonadales are enriched at the Bellairs site. Acinetobacter is a gram-negative genus which plays an important role in the mineralization of aromatic compounds within soil including marine systems, and many species can reduce nitrates to nitrites (Doughari et al. 2011). Our study highlights A. schindeleri and A. indicus, both emerging opportunistic human pathogens that can survive in many environments (Choi et al. 2012).

#####
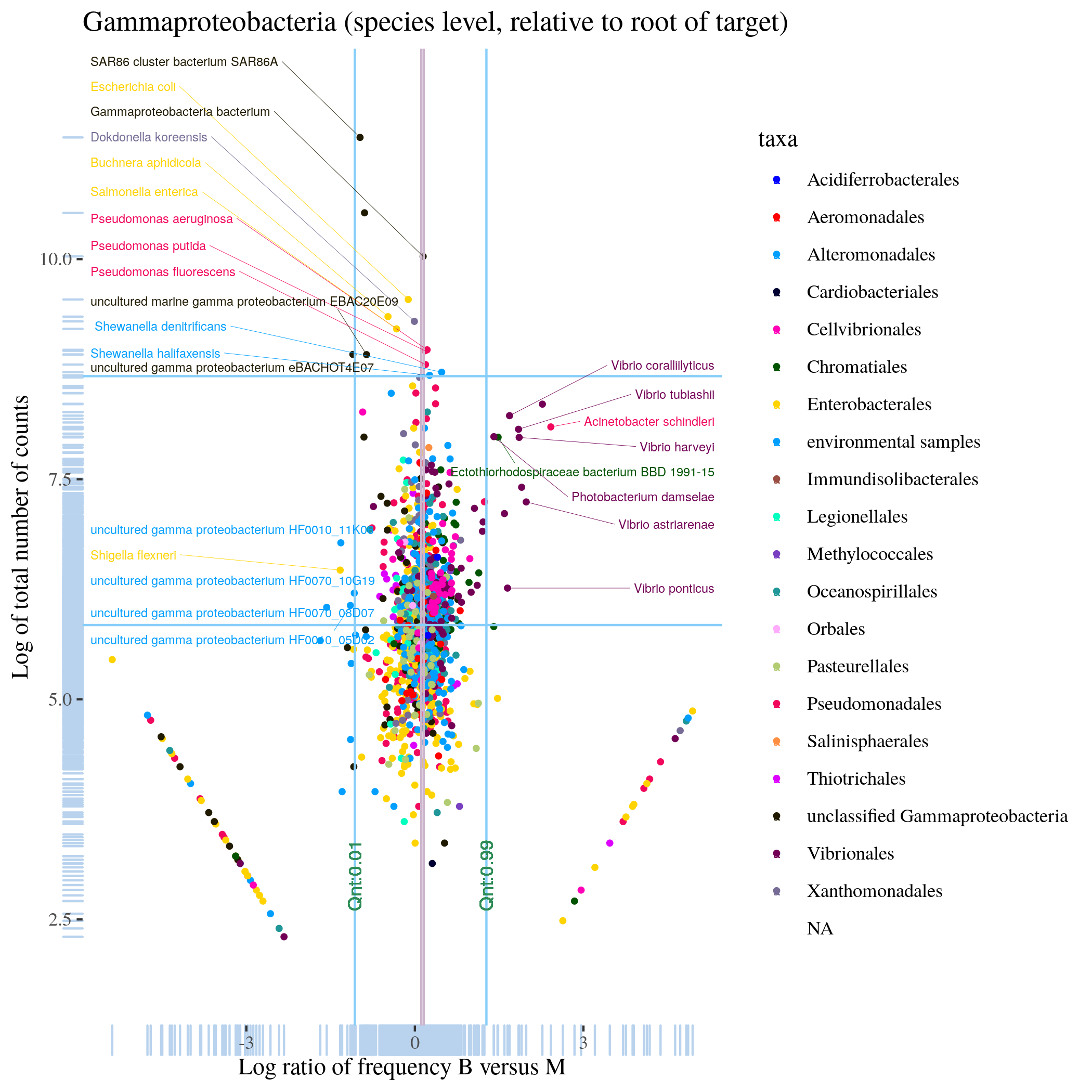


**Supplemental Figure 6.** A log-log scatter plot of the log ratio of reads for all Gammaproteobacteria species versus the total number the total number of reads for the species.

**Supplement Information 3: the Stramenopiles-Alveolates-Rhizaria (SAR) supergroup and Foraminifera**

Many species of Stramenopiles are observed uniquely at Bellairs (**Figure 3B** and **Supplemental Figure 7**). Within the Stramenopiles, several marine diatoms were identified including Licmophora (common epiphytes abundant on substrates such as filamentous algae or corals where they can form mats which smother coral (Yamashiro et al. 2012)); Cylindrotheca (ubiquitous in coastal areas worldwide that utilize latent nitrogen and phosphorus nutrients in the environment (Li et al. 2015)); Seminavis (often associated with seaweed living within coral reef ecosystems (Park et al. 2018)); Minutocellus (specifically species M. polymorphus, a symbiont of benthic Foraminifera (Schmidt et al. 2015) found to be codominant with brown tide, which thrives in eutrophic marine environments (Qiao et al. 2017)); Hyalosira (known to attach to seaweeds in intertidal environments (Totti et al. 2009)); and, Grammatophora (an epiphytic marine genus of diatoms found in coastal marine environments (Sato et al. 2008)). Furthermore, Bellairs identifies members of the genus Gyrodinium (marine heterotrophic dinoflagellates which prey on diatoms and can cause red tides (Yim et al. 2007)), and Pleurocladia (benthic brown alga epiphytes of macroalgae).

Analogous to the Stramenopiles, the Foraminifera contribute a surprising number of uniquely identified genera (9 of 34; p << 0.01, hypergeometric binned by clades directly from the root of Eukaryota; **Figure 3B**). There was no evidence of Foraminifera at Maycocks. Foraminifera are single-celled shelled protists and recognized as one of the most abundant groups of microorganisms in the shallow marine waters. The fact that their size range (100µm-20cm) is well beyond our filtered range may explain the paucity of reads for these taxa. Some amoeboid protists are often benthic or live in the sea sediment; at least 40 morphospecies are planktonic and form symbiotic relationships with marine algae. They are sensitive to the subtle changes in the ambient environment and species are known to survive and increase in numbers in polluted areas (Nigam et al. 2006). Planktonic foraminifera play an important role in the carbonate pump, contributing up to 50% of the total carbonate in the ocean sediment (Manno et al. 2012). Genera Neogloboquadrina, Globorotalia, Planoglbratella, Elphidium, Rosalina, Allogromia and Rotaliella are all recognized as planktic. Rosalina, for example, is known to attach to seaweed and other marine benthic surfaces within shallow environments and can also be found unattached within sediment (Todd 1965). Species such as R. leei are able to thrive in ecologically stressed environments (Kurtarkar Raikar et al. 2011).


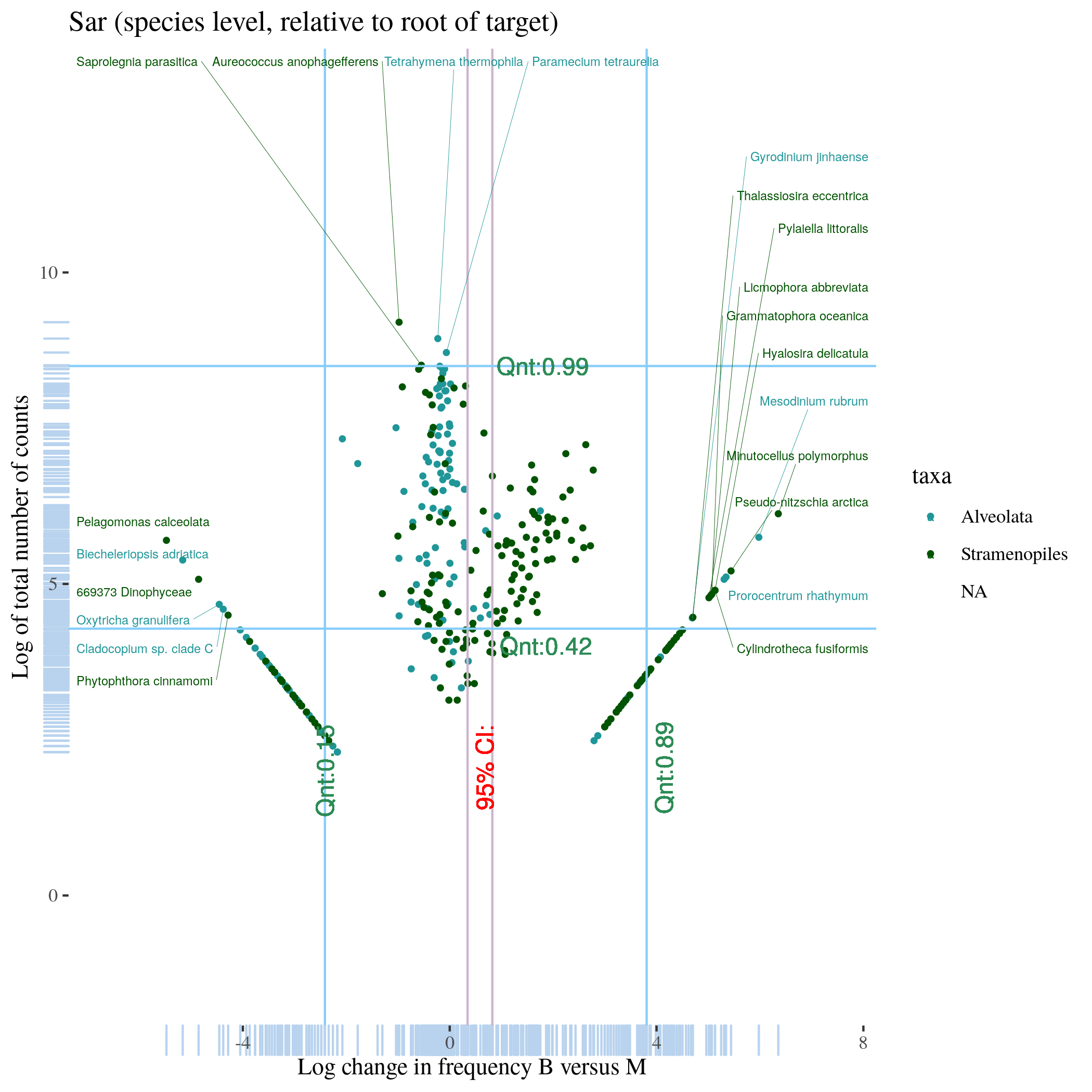


**Supplemental Figure 7.** A log-log scatter plot of the log ratio of reads for all SAR species versus the total number the total number of reads for the species.

**Supplemental Information 4: Integration with the Tara Oceans’ data**

The metagenomic sequences and the associated metadata were downloaded from the companion website of Sunagawa and colleagues (Sunagawa et al. 2015). Samples that were not filtered for organisms in the range of 0.22μm and 3μm were excluded from our analyses. With respect to the metadata, we primarily made use of Tables W1 (description of the sampling sites), W5 (miTAG related data) and W8 (a broad range of molecular concentrations, temperatures, and computational measures of diversity). The miTAG 16S abundance estimations were loaded into our R data frame as described in Methods 8. We first attempted to adjust for zeros in this dataset alongside our two samples using a multiplicate Bayesian approach (Martín-Fernández et al. 2011), but could not achieve convergence, since some Tara Oceans samples had zero counts for >50% of the taxa. To achieve convergence, we had to remove taxa, a procedure we deemed undesirable and unnecessary as the observed adjustments were very small. We opted instead to simply add 1 to all entries in the count matrix. Clustering was performed by first identifying the most abundant taxa for each sample, and then transforming these abundances to ranks. Then the Kendall τ distance metric was used with Ward’s algorithm to construct two dimensional hierarchical clusters.

Aitchison J (1986) The Statistical Analysis of Compositional Data. Springer Netherlands, Dordrecht

Amin AKMR, Feng G, Al-saari N, Meirelles PM, Yamazaki Y, Mino S, Thompson FL, Sawabe T, Sawabe T (2016) The First Temporal and Spatial Assessment of Vibrio Diversity of the Surrounding Seawater of Coral Reefs in Ishigaki, Japan. Front Microbiol 7:

Choi J-Y, Kim Y, Ko EA, Park YK, Jheong W-H, Ko G, Ko KS (2012) Acinetobacter species isolates from a range of environments: species survey and observations of antimicrobial resistance. Diagn Microbiol Infect Dis 74:177–180

Cróquer A, Bastidas C, Elliott A, Sweet M (2013) Bacterial assemblages shifts from healthy to yellow band disease states in the dominant reef coral *Montastraea faveolata*: Shifts in microbial diversity from healthy to YBD tissues. Environ Microbiol Rep 5:90–96

Doughari HJ, Ndakidemi PA, Human IS, Benade S (2011) The Ecology, Biology and Pathogenesis of *Acinetobacter* spp.: An Overview. Microbes Environ 26:101–112

Gloor GB, Macklaim JM, Pawlowsky-Glahn V, Egozcue JJ (2017) Microbiome Datasets Are Compositional: And This Is Not Optional. Front Microbiol 8:

Goarant C, Ansquer D, Herlin J, Domalain D, Imbert F, De Decker S (2006) “Summer Syndrome” in Litopenaeus stylirostris in New Caledonia: Pathology and epidemiology of the etiological agent, Vibrio nigripulchritudo. Aquaculture 253:105–113

Kurtarkar Raikar S, Nigam R, Saraswat R, N L (2011) Regeneration and abnormality in benthic foraminifera Rosalina leei: Implications in reconstructing past salinity changes. Riv Ital Paleontol E Stratigr 117:

Li J, Dong S, Tian X, Shi C, Wang F, Gao Q, Zhu C (2015) Effects of the diatom Cylindrotheca fusiformis on the growth of the sea cucumber Apostichopus japonicus and water quality in ponds. Aquac Int 23:955–965

Manno C, Morata N, Bellerby R (2012) Effect of ocean acidification and temperature increase on the planktonic foraminifer Neogloboquadrina pachyderma (sinistral). Polar Biol 35:1311–1319

Martín-Fernández JA, Palarea-Albaladejo J, Olea RA (2011) Dealing with Zeros. In: Pawlowsky-Glahn V., Buccianti A. (eds) Compositional Data Analysis. John Wiley & Sons, Ltd, Chichester, UK, pp 43–58

Munn CB (2015) The Role of Vibrios in Diseases of Corals. Microbiol Spectr 3:

Nigam R, Saraswat R, Panchang R (2006) Application of foraminifers in ecotoxicology: Retrospect, perspect and prospect. Environ Int 32:273–283

Park JS, Lobban C, Lee K-W (2018) Diatoms associated with seaweeds from Moen Island in Chuuk Lagoon, Micronesia. Phytotaxa 351:101

Qiao L, Chen Y, Mi T, Zhen Y, Gao Y, Yu Z (2017) Quantitative PCR analysis of the spatiotemporal dynamics of Aureococcus anophagefferens and Minutocellus polymorphus and the relationship between brown tides and nutrients in the coastal waters of Qinhuangdao, China. J Appl Phycol 29:297–308

Quinn TP, Erb I, Gloor G, Notredame C, Richardson MF, Crowley TM (2019) A field guide for the compositional analysis of any-omics data. GigaScience 8:giz107

Rivas AJ, Lemos ML, Osorio CR (2013) Photobacterium damselae subsp. damselae, a bacterium pathogenic for marine animals and humans. Front Microbiol 4:

Sato S, Mann D, Nagumo T, Tanaka J, Tadano T, Medlin L (2008) Auxospore Fine Structure and Variation in Modes of Cell Size Changes in Grammatophora Marina (Bacillariophyta). Phycologia 47:

Schmidt C, Morard R, Almogi-Labin A, Weinmann AE, Titelboim D, Abramovich S, Kucera M (2015) Recent Invasion of the Symbiont-Bearing Foraminifera Pararotalia into the Eastern Mediterranean Facilitated by the Ongoing Warming Trend. PLoS One San Franc 10:e0132917

Sunagawa S, Coelho LP, Chaffron S, Kultima JR, Labadie K, Salazar G, Djahanschiri B, Zeller G, Mende DR, Alberti A, Cornejo-Castillo FM, Costea PI, Cruaud C, d’Ovidio F, Engelen S, Ferrera I, Gasol JM, Guidi L, Hildebrand F, Kokoszka F, Lepoivre C, Lima-Mendez G, Poulain J, Poulos BT, Royo-Llonch M, Sarmento H, Vieira-Silva S, Dimier C, Picheral M, Searson S, Kandels-Lewis S, Tara Oceans coordinators, Bowler C, de Vargas C, Gorsky G, Grimsley N, Hingamp P, Iudicone D, Jaillon O, Not F, Ogata H, Pesant S, Speich S, Stemmann L, Sullivan MB, Weissenbach J, Wincker P, Karsenti E, Raes J, Acinas SG, Bork P, Boss E, Bowler C, Follows M, Karp-Boss L, Krzic U, Reynaud EG, Sardet C, Sieracki M, Velayoudon D (2015) Structure and function of the global ocean microbiome. Science 348:1261359–1261359

Sussman M, Mieog JC, Doyle J, Victor S, Willis BL, Bourne DG (2009) Vibrio zinc-metalloprotease causes photoinactivation of coral endosymbionts and coral tissue lesions. PloS One 4:e4511

Todd R (1965) A new Rosalina (foraminifera) parasitic on a bivalve. Deep Sea Res Oceanogr Abstr 12:831–837

Totti C, Poulin M, Romagnoli T, Perrone C, Pennesi C, De Stefano M (2009) Epiphytic diatom communities on intertidal seaweeds from Iceland. Polar Biol 32:1681–1691

Xie ZY, Hu CQ, Zhang LP, Chen C, Ren CH, Shen Q (2007) Identification and pathogenicity of Vibrio ponticus affecting cultured Japanese sea bass, Lateolabrax japonicus (Cuvier in Cuvier and Valenciennes). Lett Appl Microbiol 45:62–67

Yamashiro H, Mikame Y, Suzuki H (2012) Localized outbreak of attached diatoms on the coral Montipora due to low-temperature stress. Sci Rep 2:552

Yim JH, Kim SJ, Ahn SH, Lee HK (2007) Characterization of a novel bioflocculant, p-KG03, from a marine dinoflagellate, Gyrodinium impudicum KG03. Bioresour Technol 98:361–367
